# Impact of Peripheral Inflammation on Blood–Brain Barrier Dysfunction and Its Role in Neurodegenerative Diseases

**DOI:** 10.3390/ijms26062440

**Published:** 2025-03-09

**Authors:** Ana Isabel Beltran-Velasco, Vicente Javier Clemente-Suárez

**Affiliations:** 1 NBC Group, Psychology Department, School of Life and Nature Sciences, Nebrija University, 28248 Madrid, Spain; abeltranv@nebrija.es; 2 Faculty of Medicine, Health and Sports, Universidad Europea de Madrid, Villaviciosa de Odón, 28670 Madrid, Spain; 3 Grupo de Investigación en Cultura, Educación y Sociedad, Universidad de la Costa, Barranquilla 080002, Colombia

**Keywords:** blood–brain barrier, peripheral inflammation, gut–brain axis, gut microbiota, neurodegeneration

## Abstract

The blood–brain barrier (BBB) is essential for maintaining brain homeostasis by regulating molecular exchange between the systemic circulation and the central nervous system. However, its dysfunction, often driven by peripheral inflammatory processes, has been increasingly linked to the development and progression of neurodegenerative diseases such as Alzheimer’s and Parkinson’s. Emerging evidence suggests that the gut–brain axis plays a key role in BBB integrity, with intestinal dysbiosis and chronic inflammation contributing to barrier disruption through immune and metabolic pathways. Furthermore, the selective vulnerability of specific brain regions to BBB dysfunction appears to be influenced by regional differences in vascularization, metabolic activity, and permeability, making certain areas more susceptible to neurodegenerative processes. This review explored the molecular mechanisms linking peripheral inflammation, gut microbiota, and BBB dysfunction, emphasizing their role in neurodegeneration. A comprehensive literature review was conducted using Web of Science, PubMed, Scopus, Wiley, ScienceDirect, and Medline, covering publications from 2015 to 2025. The findings highlight a complex interplay between gut microbiota-derived metabolites, immune signaling, and BBB permeability, underscoring the need for targeted interventions such as microbiome modulation, anti-inflammatory therapies, and advanced drug delivery systems. The heterogeneity of the BBB across different brain regions necessitates the development of region-specific therapeutic strategies. Despite advancements, critical knowledge gaps persist regarding the precise mechanisms underlying BBB dysfunction. Future research should leverage cutting-edge methodologies such as single-cell transcriptomics and organ-on-chip models to translate preclinical findings into effective clinical applications. Addressing these challenges will be crucial for developing personalized therapeutic approaches to mitigate the impact of BBB dysfunction in neurodegenerative diseases.

## 1. Introduction

The blood–brain barrier (BBB) is a vital structure that plays a critical role in regulating the brain’s internal environment, thereby ensuring the stability necessary for neuronal function. Located at the interface between systemic circulation and the central nervous system (CNS), the BBB’s primary function is to control the exchange of molecules, cells, and signals between these compartments [1,2]. In recent years, there has been a surge of interest in the role of peripheral factors, particularly inflammation and gut microbiota, in modulating BBB function and contributing to its dysfunction in neurological disorders. This barrier is critical for maintaining a constant brain microenvironment, protecting neural tissue from potentially harmful external influences [3,4].

The BBB comprises specialized endothelial cells that line the cerebral capillaries. In contrast to the peripheral endothelium, these cells are devoid of fenestrations and are characterized by the presence of highly developed tight junctions composed of transmembrane proteins, including claudin-5, occludin, and zona occludens proteins (ZO-1 and ZO-2) [5,6]. These tight junctions form a highly selective physical barrier [7], restricting paracellular molecular passage and preventing the entry of toxins, pathogens, and plasma macromolecules into the brain parenchyma [8,9]. Additionally, microbiota-derived metabolites have been identified as modulators of these proteins, influencing BBB integrity.

### 1.1. Fundamental Mechanisms of BBB Dysfunction Under Inflammatory Conditions

In healthy individuals, the BBB strictly regulates the transit of substances and cells between the periphery and the CNS. However, under peripheral inflammation, its function is compromised due to the activation of multiple inflammatory pathways that alter its structure and function [10]. Recent studies have demonstrated that gut dysbiosis can exacerbate peripheral inflammation and promote BBB alterations, suggesting a direct interconnection between these factors. Chronic inflammation, triggered by bacterial or viral infections or autoimmune processes, generates an environment rich in pro-inflammatory cytokines, such as interleukin-1 beta (IL-1β), tumor necrosis factor-alpha (TNF-α), and interleukin-6 (IL-6), which directly impact cerebral endothelial cells [11,12,13].

The increase in these inflammatory mediators activates intracellular pathways in endothelial cells, mainly those mediated by the transcription factor NF-κB [14]. The activation of NF-κB induces alterations in the expression of pivotal tight junction proteins, including claudin-5 and occludin. These alterations result in the degradation of these proteins and a subsequent reduction in barrier impermeability [15,16]. This structural breakdown facilitates the passage of plasma molecules and immune cells into the brain parenchyma, promoting a neuroinflammatory state [17].

Recent studies have identified a novel mechanism by which gut microbiota influences the integrity of the BBB. Specifically, the influence of microbial metabolites, such as short-chain fatty acids (SCFAs), on the regulation of peripheral inflammation has emerged as a significant factor in BBB dysfunction [18,19,20]. Pro-inflammatory bacterial byproducts, such as lipopolysaccharide (LPS), translocate across the intestinal barrier in cases of dysbiosis, thereby weakening its integrity. Conversely, SCFAs reinforce tight junctions and modulate immune responses within the intestinal tract, thus potentially enhancing the protective barrier function of the BBB. Recent studies have identified a correlation between gut dysbiosis and increased LPS production, which activates the Toll-like receptor 4 (TLR4), triggering systemic inflammatory responses that impair BBB integrity [21,22]. Additionally, certain bacterial metabolites, such as SCFAs, have demonstrated protective effects on the BBB by reinforcing tight junctions and modulating immune responses [23,24].

### 1.2. The Gut–Brain Axis as a Modulator of the BBB

The gut–brain axis is a bidirectional communication network connecting the CNS with the gastrointestinal tract, integrating neuroendocrine, immunological, and metabolic signals [25]. A pivotal element of this interaction pertains to the gut microbiota, an ecosystem comprising microorganisms that exert influence on brain function by means of bioactive metabolites, cytokines, and neurotransmitters [12,26]. Recent research has identified a capacity for microbiota to modulate BBB function through the production of beneficial metabolites and the regulation of immune system activity and oxidative stress. Mounting evidence suggests that alterations in microbiota composition can affect the BBB, modifying its permeability and contributing to neuroinflammation and neurodegeneration [18,27].

The evidence associating peripheral inflammation with BBB dysfunction in various neurological pathologies provides a framework for understanding the mechanisms underlying neurodegeneration and underscores the importance of addressing peripheral inflammation as a pivotal therapeutic target. The identification of strategies to protect and restore BBB function offers a promising approach for the development of more effective neuroprotective therapies.

The primary objective of this review is to analyze the relationship between peripheral inflammation and BBB dysfunction, emphasizing the molecular mechanisms linking this dysfunction to neurodegenerative pathologies, and to evaluate emerging therapeutic implications for restoring BBB integrity and preventing associated neuronal damage.

In order to achieve the overarching objective, the following specific objectives were proposed: (i) examine the molecular and cellular mechanisms by which peripheral inflammation affects BBB structure and function, including tight junction dysregulation, increased permeability, and altered selective transport function; (ii) analyze evidence from studies in animal models and humans showing the relationship between chronic peripheral inflammation and neurodegenerative pathologies, highlighting diseases such as Alzheimer’s, Parkinson’s, and multiple sclerosis; (iii) review current and emerging therapeutic strategies aimed at reducing peripheral inflammation and protecting the BBB, such as anti-inflammatory interventions and the modulation of the gut microbiota; (iv) evaluate regional differences in the BBB, its susceptibility to dysfunction in various brain areas, and how this may explain the selective vulnerability of certain brain regions in neurodegenerative diseases; and (v) propose new directions for therapeutic research that integrate multidisciplinary approaches, such as the use of neuroprotective drugs targeting the BBB and personalized strategies for treatment delivery based on barrier heterogeneity.

## 2. Methodology

The present article focuses on the mechanisms by which the gut–brain axis modulates the blood–brain barrier and how dysfunction of this barrier, induced by peripheral inflammatory processes, contributes to the development and progression of various neurological pathologies. The most salient advances in the realm of research concerning the intricate relationship between intestinal inflammation, microbiota, and the BBB are meticulously examined, with a particular emphasis on the underlying molecular mechanisms and their clinical ramifications in neurodegenerative and neuroinflammatory diseases.

To ensure the quality and relevance of the studies reviewed, inclusion and exclusion criteria were established. The inclusion criteria were as follows: (i) studies specifically addressing the impact of the gut–brain axis on BBB, and (ii) investigations on the relationship between peripheral inflammation and BBB dysfunction in neurological diseases. Studies that did not explicitly address BBB dysfunction or that focused only on peripheral pathologies without exploring their influence on the CNS were excluded.

The search was conducted in the following databases: Web of Science, PubMed, Scopus, Wiley, ScienceDirect, and Medline. The search was limited to studies published between 2015 and 2025. The methodology used in previous comprehensive literature reviews was followed, ensuring a systematic and exhaustive approach [28,29].

To optimize the search and ensure comprehensive coverage, Boolean operators and combinations of key terms were used: (gut-brain axis OR intestinal inflammation OR gut microbiota) AND (blood-brain barrier OR endothelial dysfunction OR neurodegeneration OR neuroinflammation) AND (permeability OR tight junctions OR astrocytes OR therapy). Priority was given to the inclusion of comprehensive review articles, relevant clinical studies, and experimental work that provided significant data on the topic.

This methodological approach facilitated the compilation of a substantial and contemporary scientific literature base, providing a foundation for the detailed examination of the mechanisms underlying BBB dysfunction and the potential novel therapeutic strategies aimed at preserving its integrity within the context of chronic peripheral inflammation.

## 3. Peripheral Inflammation as a BBB Disruptor

In healthy individuals, the BBB maintains selective permeability, regulating the movement of substances and immune cells between the peripheral circulation and the CNS, thereby preserving neuronal homeostasis. However, chronic peripheral inflammation can disrupt this selective barrier by activating inflammatory pathways that compromise BBB integrity. Emerging evidence suggests that gut microbiota dysbiosis exacerbates systemic inflammation, further impairing BBB function by altering endothelial cell signaling and promoting immune cell infiltration [30,31].

The release of pro-inflammatory cytokines, such as tumor necrosis factor-alpha (TNF-α), interleukin-6 (IL-6), and interleukin-1 beta (IL-1β), occurs in response to peripheral inflammation, leading to the activation of endothelial cells within the BBB [32]. This activation initiates intracellular signaling cascades that result in the degradation of tight junction proteins, including claudin-5 and occludin, thereby increasing permeability [33,34]. Furthermore, microbial-derived molecules, such as lipopolysaccharides (LPSs), have been demonstrated to exacerbate this process by directly stimulating Toll-like receptor 4 (TLR4) on endothelial cells, intensifying the inflammatory response and weakening barrier function [35,36,37].

Oxidative stress is another significant contributor to the disruption of the BBB in inflammatory conditions. Excessive reactive oxygen species (ROS) generated by activated endothelial cells and infiltrating immune cells impair endothelial integrity by oxidizing lipids and proteins that are critical for BBB function [38]. Recent studies have identified gut-derived metabolites, such as SCFAs, as potential regulators of oxidative stress and BBB stability. Butyrate, an SCFA, has been shown to reinforce tight junctions, thereby exerting a protective effect on the BBB. However, an imbalance in gut microbial composition, which can be triggered by various factors, may lead to a reduction in SCFA availability, thus increasing the susceptibility of the BBB to oxidative damage [39,40].

Another key mechanism by which peripheral inflammation disrupts the BBB is the upregulation of adhesion molecules such as intercellular adhesion molecule-1 (ICAM-1) and vascular cell adhesion molecule-1 (VCAM-1) on endothelial cells [41]. These molecules facilitate the adhesion and transendothelial migration of immune cells into the CNS, a process particularly well documented in multiple sclerosis and other neuroinflammatory disorders [42]. Notably, dysbiosis-induced systemic inflammation has been implicated in the upregulation of these adhesion molecules, further exacerbating immune cell infiltration into the brain [43,44].

The impact of peripheral inflammation on the BBB is multifaceted, extending beyond increased permeability. Chronic inflammation also alters BBB transporter function, affecting nutrient and metabolite exchange necessary for neuronal survival [45]. In this line, the process of the inflammation-induced downregulation of glucose transporter 1 (GLUT1) has been demonstrated to reduce glucose availability in the brain. This, in turn, has the potential to impair cognitive function and accelerate neurodegenerative processes [46]. Similarly, alterations in amino acid and peptide transport systems have been shown to contribute to disruptions in neurotransmitter balance, exacerbating neuronal damage [47].

**Figure 1:**
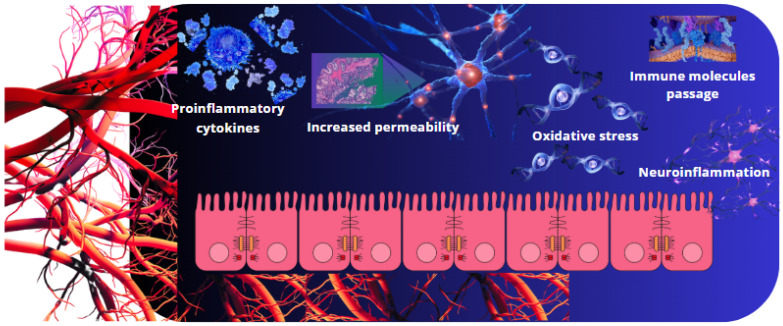
The mechanisms underlying BBB dysfunction induced by peripheral inflammation are multifaceted. Pro-inflammatory mediators activate intracellular pathways in brain endothelial cells, resulting in the degradation of tight junction proteins and an increase in adhesion molecule expression. This heightened oxidative stress and reduced transport of essential nutrients contribute to BBB disruption and the subsequent development of neuroinflammation.

Recent studies have established strong links between peripheral inflammation, gut microbiota, and BBB dysfunction in the pathogenesis of neurodegenerative diseases [48]. In Alzheimer’s disease, chronic systemic inflammation facilitates β-amyloid deposition through BBB impairment, while in Parkinson’s disease, heightened BBB permeability in the substantia nigra promotes α-synuclein aggregation and dopaminergic neuron loss [49,50]. These findings underscore the importance of targeting systemic inflammation and gut microbiota in therapeutic strategies aimed at preserving BBB integrity and preventing neurodegenerative progression (Figure 1).

## 4. BBB Heterogeneity and Regional Differences in Neuropathologies

The BBB is a highly specialized structure that plays a critical role in maintaining brain homeostasis by regulating nutrient entry and protecting against harmful substances. However, it is important to note that the BBB exhibits heterogeneity across different brain regions, with variations in structure and function attributable to anatomical and physiological characteristics specific to each area [51]. Recent evidence suggests that this heterogeneity influences regional susceptibility to inflammatory and neurodegenerative processes, particularly through interactions with systemic inflammation and gut microbiota-derived metabolites [52,53]. These disparities in vascular organization, in conjunction with region-specific BBB properties, have been demonstrated to exert a substantial influence on the vulnerability of gray and white matter to neuropathologies, including Alzheimer’s and Parkinson’s diseases.

### 4.1. Vascularization and Susceptibility to BBB Dysfunction in Gray and White Matter

The brain’s vascularization is highly differentiated to meet the metabolic needs of distinct areas. Gray matter, which contains a high density of neuronal bodies, is richly vascularized, ensuring a constant supply of oxygen and nutrients required for neuronal function [54,55]. The BBB in gray matter is characterized by tightly regulated endothelial junctions, which provide an effective barrier against pathogens, toxins, and plasma macromolecules [56]. However, recent studies have indicated that gut microbiota can modulate these endothelial cells via microbial metabolites, thereby altering BBB permeability in a region-dependent manner [57,58]. Conversely, white matter, which is composed primarily of myelinated axonal fibers, has a lower density of blood vessels and a more diffuse vascular architecture, reflecting its comparatively lower metabolic demands [59,60]. This reduced vascularization renders the BBB in white matter more vulnerable to permeability changes under ischemic or inflammatory conditions. Recent findings indicate that systemic inflammation and microbiota dysbiosis can exacerbate white matter vulnerability by increasing endothelial stress, altering nutrient transport, and promoting immune cell infiltration [2]. These factors may explain the selective involvement of white matter in neuroinflammatory diseases such as multiple sclerosis.

In addition, regional disparities in BBB structure and function influence the propensity to neurodegeneration. In gray matter, the BBB’s heightened regulation is paramount for maintaining neuronal homeostasis and synaptic activity. Conversely, white matter endothelial junctions exhibit reduced restriction, potentially enabling the inadvertent passage of inflammatory molecules and immune cells, particularly during pathological states. This differential permeability may contribute to region-specific disease progression in neurodegenerative conditions [61,62].

### 4.2. Selective Vulnerability of Specific Cerebral Regions in Neurodegenerative Diseases

The BBB’s heterogeneity contributes to the selective vulnerability of specific brain regions to neurodegenerative diseases, as evidenced by conditions such as Alzheimer’s and Parkinson’s disease. These conditions illustrate how BBB dysfunction influences disease progression by facilitating the entry of inflammatory mediators, toxins, and misfolded proteins [63,64]. In the field of neurodegenerative diseases, several hypotheses have been postulated to account for the selective vulnerability of particular brain regions. A prominent theory posits that metabolic demands and oxidative stress render specific areas more susceptible to neurodegeneration. Regions such as the hippocampus and substantia nigra, which exhibit high energy expenditure and extensive synaptic connectivity, are hypothesized to be particularly vulnerable to inflammatory and metabolic alterations [25,64].

Alzheimer’s disease, a prototypical example of neurodegeneration, is characterized by the accumulation of toxic proteins, such as β-amyloid, which predominantly aggregates in the entorhinal cortex and hippocampus, areas crucial for memory and learning [65]. The BBB in these regions, due to its high vascularization, is particularly exposed to inflammatory mediators and metabolic dysregulation arising from imbalances in the gut microbiota [66]. The dysfunction of BBB transport mechanisms and the destabilization of tight junctions facilitate the passage of β-amyloid from the peripheral circulation into the brain, accelerating plaque formation and exacerbating neurodegeneration [67]. Additionally, chronic peripheral inflammation in Alzheimer’s disease further compromises BBB function, allowing increased immune cell infiltration and exacerbating neuroinflammation in these key regions [68,69].

In Parkinson’s disease, the substantia nigra, which contains dopaminergic neurons responsible for motor control, exhibits heightened susceptibility to neurodegeneration [70]. Despite its relatively high vascularization, the BBB in the substantia nigra has been shown to have increased permeability, potentially facilitating the entry of inflammatory agents and reactive oxygen species (ROS) [71]. Recent evidence suggests that gut microbiota dysregulation may contribute to increased BBB permeability in the substantia nigra, promoting α-synuclein aggregation and neuronal damage [72]. This BBB dysfunction could permit the passage of neurotoxic proteins from the peripheral circulation, exacerbating dopaminergic neuron loss and accelerating the progression of Parkinsonian symptoms [73,74].

Neuroinflammatory diseases affecting white matter, such as multiple sclerosis, present a distinct pattern of BBB dysfunction, with the endothelial barrier in white matter being particularly susceptible to inflammatory processes, allowing immune cell infiltration, including T lymphocytes and monocytes [75]. This immune cell influx leads to myelin degradation, disrupting efficient neuronal signaling and resulting in neurological deficits [76]. Recent studies have indicated that gut microbiota-derived inflammatory signals may contribute to white matter BBB breakdown, thereby enhancing vulnerability to immune-mediated demyelination [77,78].

**Figure 2:**
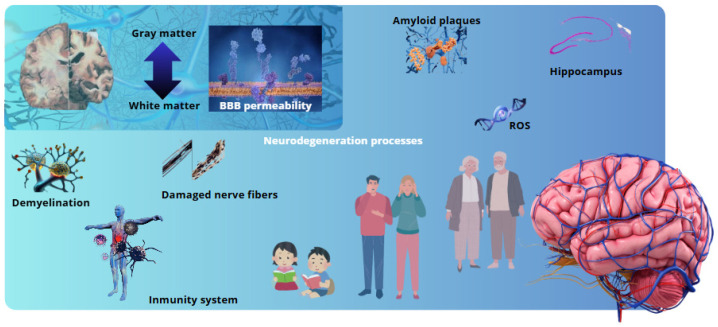
The BBB exhibits heterogeneity across different brain regions. This heterogeneity is characterized by differences in the permeability of the BBB in the white matter and gray matter, as well as the vulnerability of these regions to common neurodegenerative processes in pathologies such as Alzheimer’s and multiple sclerosis.

The present findings suggest that the selective vulnerability of specific brain regions to neurodegeneration may be influenced by a combination of intrinsic factors, such as metabolic demand and oxidative stress, and systemic inflammatory signals modulated by the gut microbiota. The hippocampus and substantia nigra, for instance, appear to be particularly susceptible to inflammatory insults, likely due to a combination of high vascularization, metabolic activity, and increased permeability of the BBB under inflammatory conditions. In this context, microbial-derived components such as lipopolysaccharides have been shown to exacerbate neuroinflammatory processes, while SCFAs have been demonstrated to exert a modulatory role in preserving BBB integrity. This observed regional heterogeneity in BBB susceptibility underscores the necessity to consider gut–brain interactions as a potential factor in explaining the distinct patterns of neurodegeneration observed across different disorders (Figure 2).

## 5. Peripheral Inflammation and Neurodegeneration

Chronic peripheral inflammation has been identified as a significant contributor to the progression of neurodegenerative diseases, given its capacity to disrupt CNS homeostasis and exacerbate neuronal damage. Mounting evidence indicates that systemic inflammatory responses, particularly those associated with gut microbiota dysbiosis, play a crucial role in neurodegeneration by impairing blood–brain barrier integrity, activating microglia, and promoting the accumulation of neurotoxic proteins [79,80]. The interplay between peripheral inflammation and neurodegeneration involves a complex cascade of immune activation, oxidative stress, and metabolic dysregulation that accelerates disease pathology [12,81].

### 5.1. Microglial Activation and Oxidative Stress

Peripheral inflammation has been demonstrated to induce neurodegeneration, a process that is associated with the sustained activation of microglia, the resident immune cells of the CNS. Inflammatory cytokines, such as tumor necrosis factor-alpha (TNF-α), interleukin-6 (IL-6), and IL-1β, which are elevated in chronic systemic inflammation, have been shown to promote microglial activation and shift them toward a pro-inflammatory phenotype [82,83]. This sustained activation of microglia has been shown to lead to the increased production of ROS and nitric oxide, which in turn causes oxidative stress, synaptic dysfunction, and neuronal apoptosis [84]. Furthermore, systemic inflammation has been demonstrated to impair the function of the BBB, thereby allowing the entry of peripheral immune cells and inflammatory mediators into the CNS, which in turn exacerbates neuroinflammation and neuronal injury [85].

### 5.2. Peripheral Inflammation and β-Amyloid Pathology in Alzheimer’s Disease

In Alzheimer’s disease, chronic inflammation and BBB dysfunction have been implicated in the accumulation of β-amyloid (Aβ) plaques. Systemic inflammation weakens Aβ clearance mechanisms, leading to its accumulation in vulnerable brain regions, such as the hippocampus [62,86]. Furthermore, the entry of peripheral inflammatory mediators through a compromised BBB exacerbates tau pathology, contributing to neuronal loss and cognitive decline [87]. Recent studies have indicated that gut microbiota alterations may modulate Aβ pathology by influencing peripheral immune responses and inflammatory signaling. For instance, recent research has shown that changes in gut microbial composition can alter systemic cytokine levels, impacting Aβ deposition and clearance mechanisms in the brain [88,89]. This observation suggests the potential involvement of a gut–brain axis in the pathogenesis of Alzheimer’s disease, whereby gut dysbiosis contributes to systemic inflammation, affecting blood–brain barrier (BBB) permeability and accelerating neurodegenerative changes [90].

### 5.3. Peripheral Inflammation and α-Synuclein Aggregation in Parkinson’s Disease

Parkinson’s disease exacerbates α-synuclein aggregation, a hallmark of PD pathology. Unlike in Alzheimer’s disease, where β-amyloid accumulation is influenced by BBB dysfunction, in PD, systemic inflammation particularly impacts dopaminergic neurons by increasing oxidative stress and disrupting mitochondrial function. Increased BBB permeability in the substantia nigra has been associated with enhanced immune cell infiltration, oxidative damage, and dopaminergic neuron loss [91,92]. Furthermore, gut microbiota alterations in PD patients have been linked to increased levels of pro-inflammatory cytokines and LPS, which may further compromise BBB integrity and promote α-synuclein misfolding and aggregation [93]. This highlights the role of systemic inflammation and microbial metabolites in driving neurodegenerative mechanisms beyond the CNS.

### 5.4. Metabolic and Neurotransmission Alterations

Peripheral inflammation has been demonstrated to exacerbate protein aggregation, in addition to altering brain metabolism and neurotransmission. Pro-inflammatory cytokines have been shown to interfere with synaptic plasticity by impairing glutamate homeostasis and reducing neurotrophic support, leading to cognitive dysfunction and motor deficits [94]. Furthermore, metabolic disruptions induced by systemic inflammation, such as impaired glucose metabolism and mitochondrial dysfunction, have been observed in both Alzheimer’s disease and Parkinson’s disease, further exacerbating neuronal vulnerability [95,96].

### 5.5. Therapeutic Implications

Current therapeutic strategies for neurodegenerative diseases principally concentrate on the management of symptoms rather than on the underlying mechanisms that drive disease progression. There are significant unmet needs in the effective modulation of neuroinflammation and BBB dysfunction, particularly in the context of systemic inflammatory responses [97]. Emerging evidence suggests that targeting systemic inflammation and modulating gut microbiota composition may offer therapeutic potential in neurodegenerative diseases. Interventions such as anti-inflammatory treatments, probiotic supplementation, and dietary modifications aimed at restoring gut microbial balance have shown promise in mitigating neuroinflammation and preserving BBB function [98]. These strategies, still in early stages, may provide novel approaches to slow disease progression and protect neuronal integrity by addressing inflammation at its systemic origin.

## 6. Therapeutic Approaches

The treatment of neurodegenerative diseases associated with BBB dysfunction and neuroinflammation poses a substantial challenge. However, recent research has identified several novel therapeutic strategies that aim to protect the BBB, regulate peripheral inflammation, and enhance drug delivery to the brain. These strategies, which are informed by research related to systemic inflammation and gut microbiota modulation, hold considerable promise in addressing the multifaceted challenges posed by neurodegenerative diseases.

### 6.1. Molecular Mechanisms That Link Inflammation-Induced BBB Dysfunction to Neurodegeneration

Chronic peripheral inflammation is widely regarded as a pivotal element in the pathophysiology of BBB dysfunction. This condition, characterized by the influx of inflammatory mediators and immune cells into the brain, has been implicated in the exacerbation of neuroinflammation and neuronal damage. Thus, anti-inflammatory interventions have emerged as a promising therapeutic approach, offering a potential means to safeguard the BBB and mitigate neurodegeneration.

A particularly salient strategy in this regard involves the use of cytokine inhibitors, which have demonstrated considerable potential with regard to neurodegenerative diseases. A growing body of research has indicated that the inhibition of pro-inflammatory cytokines can result in a reduction in the permeability of the BBB and a concomitant protection against neuronal damage [99,100]. TNF-α inhibitors, such as etanercept and adalimumab, which are already employed in the treatment of autoimmune diseases like rheumatoid arthritis, are being evaluated in clinical trials for the treatment of multiple sclerosis and Alzheimer’s [101,102]. Similarly, corticosteroids and other immunosuppressive medications have demonstrated the capacity to decrease systemic inflammation and, in certain instances, alleviate the consequences of BBB dysfunction [103].

Another relevant strategy is the modulation of intracellular signaling pathways implicated in inflammation. In this line, the nuclear factor kappa B pathway, a major regulatory pathway, is involved in the expression of various cytokines and the destabilization of tight junctions in the BBB [104]. One potential therapeutic strategy to consider is the use of molecules that inhibit NF-κB signaling [105]. Curcumin is an example of a natural compound that possesses anti-inflammatory properties [106,107]. However, other compounds, including JAK-STAT inhibitors, are being examined for their potential to reduce inflammation and safeguard the BBB [108].

### 6.2. Modulation of the Intestinal Microbiota as a Neuroprotective Strategy

In recent years, the concept of the gut–brain connection has gained significant traction within the field of neurodegenerative diseases. The gut microbiota plays a pivotal role in regulating systemic inflammation and modulating the immune response. This regulatory function can directly influence the function of the BBB [109]. It has been demonstrated that gut dysbiosis can trigger systemic inflammation, which in turn can affect the BBB and contribute to neuroinflammation. Thus, the modulation of the gut microbiota through the administration of probiotics, prebiotics, and selective antibiotics has been postulated as a prospective therapeutic modality to enhance the function of the BBB and mitigate the risk of neurodegeneration.

Probiotics, such as *Lactobacillus* and *Bifidobacterium*, have been utilized to restore balance to the gut microbiota and reduce systemic inflammation [110]. Some studies have demonstrated that the administration of probiotics in animal models of Alzheimer’s and Parkinson’s can enhance the integrity of the BBB, reduce neuroinflammation, and mitigate the accumulation of toxic proteins, including β-amyloid and α-synuclein [111]. A similar mechanism may underlie the effects of prebiotics, which are known to stimulate the proliferation of beneficial bacteria [112,113].

### 6.3. Recent Pharmacological Approaches Are Targeting BBB Heterogeneity

A significant challenge in the treatment of neurodegenerative diseases is the difficulty in effectively delivering drugs to the brain due to the restrictive function of the BBB. However, the heterogeneity of the BBB in different brain regions presents an opportunity to develop more precise and specific therapeutic approaches.

BBB vascularization and permeability have been shown to vary significantly between different brain regions, with some areas being more vulnerable to BBB dysfunction than others. The hippocampus exhibits heightened vulnerability to toxic protein accumulation and BBB dysfunction in diseases such as Alzheimer’s [114]. Similarly, in Parkinson’s disease, the substantia nigra, due to its high vascular density and the presence of dopaminergic cells, demonstrates increased susceptibility to neuroinflammation and disruption of the BBB [115].

**Figure 3:**
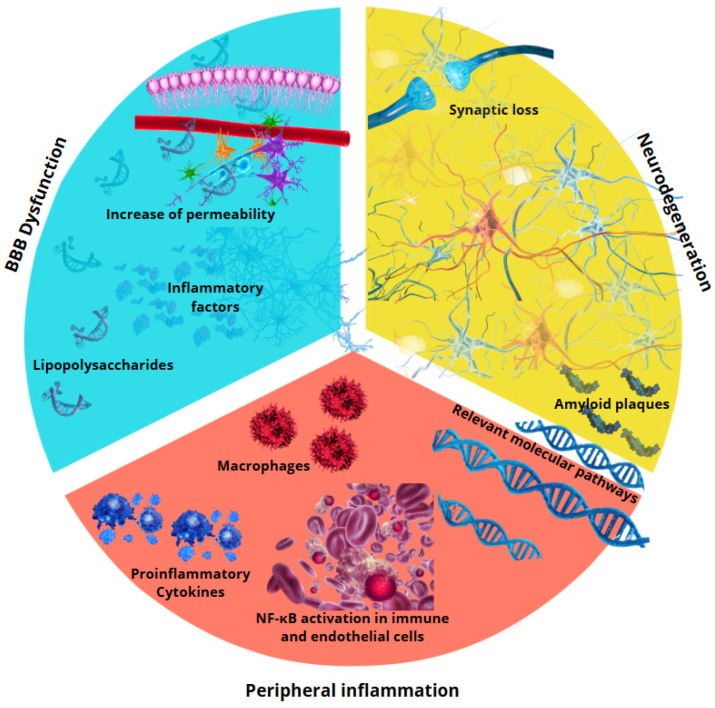
Relationship between peripheral inflammation and neurodegeneration. Peripheral inflammation, brought about by pro-inflammatory cytokines and immune cell activation, results in the dysfunction of the BBB. This dysfunction enables the permeation of noxious molecules into the brain, thereby instigating neuroinflammation and contributing to neuronal degeneration, the accumulation of pathological proteins, and synaptic loss. These processes are hallmarks of diseases such as Alzheimer’s and Parkinson’s disease.

In this line, research endeavors have focused on the development of nanoparticles and lipid vectors. These particles can be engineered to selectively target areas of the brain exhibiting BBB dysfunction [116]. This approach facilitates the efficient transport of neuroprotective drugs, while concomitantly circumventing systemic side-effects [117]. Additionally, investigations are underway that explore the use of monoclonal antibodies [118]. These antibodies are designed to bind to particular receptors present on BBB endothelial cells [119]. The goal is to employ this mechanism as a strategy to enable the selective facilitation of drug penetration into the brain (Figure 3).

## 7. Limitations and Future Research Directions

Despite the valuable insights provided by this study on the intricate relationship between the BBB and neuroinflammatory processes, several limitations must be acknowledged. First, this study primarily relies on preclinical models and observational human data, which may not comprehensively capture the complexities of BBB dysfunction across diverse neurological disorders. Future research should focus on longitudinal studies and interventional trials to establish causality and assess the efficacy of potential therapeutic interventions. Another critical limitation is the inherent heterogeneity of the BBB across different brain regions, which complicates the generalization of findings. Variations in BBB permeability and receptor expression, particularly in regions such as the hippocampus and substantia nigra, underscore the need for region-specific therapeutic strategies. Future studies should leverage advanced imaging techniques and single-cell transcriptomics to achieve a more precise characterization of BBB dynamics in both health and disease states.

Additionally, while this study highlights the potential role of the gut microbiota in modulating BBB integrity, the underlying mechanisms remain incompletely understood. Current evidence suggests that microbial metabolites influence BBB permeability through immune and metabolic pathways. Future investigations should explore the therapeutic potential of microbiome-based interventions, such as probiotics and dietary modifications, to strengthen BBB function and mitigate neuroinflammation. Finally, this study’s reliance on experimental models with limited translational applicability presents another challenge. The adoption of advanced in vitro BBB models, such as organ-on-chip systems, could provide more physiologically relevant insights into BBB dysfunction and its therapeutic modulation. Utilizing such models can bridge the gap between preclinical research and clinical applications, facilitating the development of targeted interventions. By addressing these limitations and pursuing new research directions, future studies can significantly enhance our understanding of BBB-related pathologies and contribute to the development of more effective, personalized therapeutic strategies for neurodegenerative diseases.

## 8. Practical Applications

The findings of this study have significant practical applications in both clinical and translational neuroscience, offering potential strategies for the prevention and management of neurodegenerative diseases through targeted interventions aimed at preserving BBB integrity.

(a) Early detection and biomarker development

Understanding the molecular mechanisms underlying BBB dysfunction facilitates the identification of specific biomarkers indicative of early neurodegeneration. Peripheral inflammatory markers, gut microbiota-derived metabolites, and BBB permeability indicators could serve as diagnostic tools for the early detection of conditions such as Alzheimer’s and Parkinson’s disease. The incorporation of these biomarkers into routine clinical screening could enable timely intervention and slow disease progression.

### Personalized Therapeutic Strategies

Insights into the regional heterogeneity of BBB dysfunction highlight the need for precision medicine approaches. By tailoring interventions based on an individual’s specific BBB permeability profile and inflammatory status, clinicians could optimize treatments, using region-specific drug delivery systems and personalized anti-inflammatory regimens to mitigate neurodegenerative damage.

(b) Gut modulation as an adjunct therapy

Given the established link between gut dysbiosis and BBB integrity, dietary interventions and probiotic supplementation present practical approaches to support brain health. Targeting the gut–brain axis through microbiome modulation could reduce systemic inflammation and reinforce BBB function, offering a non-invasive strategy to complement pharmacological treatments.

(c) Development of pharmacological agents

This study’s findings underscore the importance of developing drugs capable of crossing the BBB without compromising its function. Novel therapeutic agents, including nanoparticle-based drug delivery systems and monoclonal antibodies targeting BBB transport mechanisms, hold promise for enhancing drug efficacy while minimizing systemic side-effects.

(d) Lifestyle interventions

Modifiable lifestyle factors such as diet, physical activity, and stress management can play a crucial role in preserving BBB integrity. Public health initiatives aimed at promoting healthy lifestyle choices could contribute to the prevention of neuroinflammation-related BBB dysfunction, thereby reducing the incidence of neurodegenerative diseases.

(e) Integration into clinical practice guidance

The emerging evidence on the gut–brain–BBB axis and inflammation-driven neurodegeneration provides a foundation for updating clinical guidelines to incorporate BBB-protective strategies. Recommendations could include routine gut microbiota assessments, inflammation control strategies, and targeted therapeutic approaches for at-risk populations.

By translating these research findings into practical healthcare, professionals and policymakers can work towards developing comprehensive strategies to address BBB dysfunction and its role in neurodegenerative diseases, ultimately improving patient outcomes and quality of life.

## 9. Conclusions

The BBB plays a pivotal role in safeguarding the brain and preserving its homeostasis, functioning as a highly selective interface between the systemic circulation and the central nervous system. However, BBB dysfunction, frequently induced by peripheral inflammatory processes, is a pivotal factor in the development and progression of multiple neurodegenerative diseases, including Alzheimer’s, Parkinson’s, and multiple sclerosis.

One of the key conclusions of this study is the demonstration that persistent inflammation augments both the permeability of the BBB and its selective transport capacity. The consequence of this augmentation is the compromise in the supply of essential nutrients, which results in exacerbated metabolic imbalance in the brain. The impact of this BBB dysfunction is particularly pronounced in brain areas with high vascular density and greater vulnerability to pro-inflammatory agents, such as the hippocampus in Alzheimer’s disease and the substantia nigra in Parkinson’s disease.

Disruption of the BBB, which is often precipitated by inflammatory mechanisms, has been associated with an increased susceptibility to neurodegeneration. This underscores the importance of addressing peripheral inflammation when devising novel neuroprotective therapies. Research into anti-inflammatory approaches, such as cytokine inhibitors and gut microbiota modulation, has shown promise in restoring BBB integrity and preventing neuronal damage. Furthermore, advances in understanding BBB heterogeneity and its regional variability offer new opportunities to develop personalized therapies that target specific areas of the brain affected by barrier dysfunction.

A foundational element for advancement in this domain is a multidisciplinary approach that integrates advances in immunology, neuroscience, pharmacology, and biotechnology. Collaboration between researchers from diverse disciplines will facilitate the identification of biomarkers of BBB dysfunction, the development of drugs aimed at barrier protection, and the design of new drug delivery strategies that maximize therapeutic efficacy. Moreover, the exploration of combination therapies, which address both peripheral inflammation and BBB dysfunction, holds great promise in improving the treatment of chronic neurodegenerative diseases and potentially decelerating their progression.

Peripheral inflammation has been demonstrated to play a pivotal role in the dysfunction of the BBB and the development of neurodegenerative diseases. As research in this field continues to advance, it becomes increasingly imperative that therapeutic interventions prioritize the restoration of BBB function and the regulation of systemic inflammation to avert neuronal damage. The pursuit of therapeutic innovation necessitates a multidisciplinary approach, enabling the comprehensive addressing of these challenges and the promotion of more efficacious and personalized neuroprotective strategies for patients.

## Data Availability

Not applicable.
